# Reliance on emotion promotes belief in fake news

**DOI:** 10.1186/s41235-020-00252-3

**Published:** 2020-10-07

**Authors:** Cameron Martel, Gordon Pennycook, David G. Rand

**Affiliations:** 1 Sloan School of Management, Massachusetts Institute of Technology, Cambridge, USA; 2 Hill/Levene Schools of Business, University of Regina, Regina, Canada; 3 Department of Brain and Cognitive Sciences, Massachusetts Institute of Technology, Cambridge, USA

**Keywords:** Fake news, Misinformation, Dual-process theory, Emotion, Reason

## Abstract

What is the role of emotion in susceptibility to believing fake news? Prior work on the psychology of misinformation has focused primarily on the extent to which reason and deliberation hinder versus help the formation of accurate beliefs. Several studies have suggested that people who engage in more reasoning are less likely to fall for fake news. However, the role of reliance on emotion in belief in fake news remains unclear. To shed light on this issue, we explored the relationship between experiencing specific emotions and believing fake news (Study 1; *N* = 409). We found that across a wide range of specific emotions, heightened emotionality at the outset of the study was predictive of greater belief in fake (but not real) news posts. Then, in Study 2, we measured and manipulated reliance on emotion versus reason across four experiments (total *N* = 3884). We found both correlational and causal evidence that reliance on emotion increases belief in fake news: self-reported use of emotion was positively associated with belief in fake (but not real) news, and inducing reliance on emotion resulted in greater belief in fake (but not real) news stories compared to a control or to inducing reliance on reason. These results shed light on the unique role that emotional processing may play in susceptibility to fake news.

## Introduction

The 2016 US presidential election and UK Brexit vote focused attention on the spread of “fake news” (“fabricated information that mimics news media content in form but not in organizational process or intent”; Lazer et al. 2018, p. 1094) via social media. Although the fabrication of ostensible news events has been around in media such as tabloid magazines since the early twentieth century (Lazer et al. 2018), technological advances and the rise of social media provide opportunity for anyone to create a website and publish fake news that might be seen by many thousands (or even millions) of people.

The threat of misinformation is perhaps most prevalent and salient within the domain of politics. For example, within the 3 months prior to the US election, estimates indicate that fake news stories favoring Trump were shared approximately 30 million times on Facebook, while those favoring Clinton were shared approximately 8 million times (Allcott and Gentzkow 2017). Furthermore, a recent analysis suggests that, among news stories fact-checked by independent fact-checking organizations, false stories spread farther, faster, and more broadly on Twitter than true stories, with false political stories reaching more people in a shorter period of time than all other types of false stories (Vosoughi et al. 2018). These fake news stories are not only spread, but are also often believed to be true (Silverman and Singer-Vine 2016). And, in fact, merely being exposed to a fake news headline increases later belief in that headline (Pennycook et al. 2018).

Some recent studies have, in contrast, suggested that fears over widespread exposure to and consumption of fake news may be overstated, as fake news accounts for less than half a percent of Americans’ daily media diet (Allen et al. 2020). However, while similar findings have supported the conclusion that fake news websites make up a small proportion of media diets overall, these studies have also shown that fake news is disproportionately visited by specific groups of people (e.g., supporters of Donald Trump; Guess et al. 2020; social media users over the age of 65; Guess et al. 2019). Thus, regardless of the impact of fake news on the average Americans’ overall media consumption, fake news may still impact the belief in and spread of news in key political and demographic communities.

Here, we explore the psychology underlying belief in blatantly false (and implausible) news stories. In particular, we focus on the role of emotional processing in such (mis)belief.

### Motivated cognition versus classical reasoning

From a theoretical perspective, what role might we expect emotion to play? One popular perspective on belief in misinformation, which we will call the *motivated cognition account*, argues that analytic thinking—rather than emotional responses—are primarily to blame (Kahan 2017). By this account, people reason like lawyers rather than scientists, using their reasoning abilities to protect their identities and ideological commitments rather than to uncover the truth (Kahan 2013). Thus, our reasoning abilities are hijacked by partisanship, and therefore those who rely more on reasoning are better able to convince themselves of the truth of false stories that align with their ideology. This account is supported by evidence that people who engage in more analytic thinking show more political polarization regarding climate change (Kahan et al. 2012; see also Drummond and Fischhoff 2017), gun control (Kahan et al. 2017; see also Ballarini and Sloman 2017; Kahan and Peters 2017), and selective exposure to political information (Knobloch-Westerwick et al. 2017).

An alternative perspective, which we will call the *classical reasoning account*, argues that reasoning and analytic thinking do typically help uncover the truth of news content (Pennycook and Rand 2019a). By this account, individuals engaging in reasoning and reflection are less likely to mistake fake news as accurate. And, by extension, misinformation often succeeds when individuals fail to utilize reason and analytic thinking. The classical reasoning account fits within the tradition of dual-process theories of judgment, in which analytic thinking (rather than relying on “gut feelings”) is thought to often (but not always) support sound judgment (Evans 2003; Stanovich 2005). Recent research supports this account as it relates to fake news by linking the propensity to engage in analytic thinking with skepticism about epistemically suspect beliefs (Pennycook et al. 2015a, b; however, this association may be specific to Western individuals and moderated as a function of culture; see Majima et al. 2020; also see Bahçekapılı and Yilmaz 2017), such as paranormal and superstitious beliefs (Pennycook et al. 2012), conspiracy beliefs (Swami et al. 2014), delusions (Bronstein et al. 2019), and pseudo-profound bullshit (Pennycook et al. 2015a, b). Of most direct relevance, people who were more willing to think analytically when given a set of reasoning problems were less likely to erroneously believe fake news articles regardless of their partisan alignment (Pennycook and Rand 2019a), and experimental manipulations of deliberation yield similar results (Bagò et al. 2020). Moreover, analytic thinking is associated with lower trust in fake news sources (Pennycook and Rand 2019b) and less sharing of links to low quality sources on Twitter (Mosleh et al. 2020). Belief in fake news has also been associated with dogmatism, religious fundamentalism, and reflexive (rather than active/reflective) open-minded thinking (Bronstein et al. 2019; Pennycook and Rand 2019c). A recent experiment has even shown that encouraging people to think deliberately, rather than intuitively, decreased self-reported likelihood of “liking” or sharing fake news on social media (Effron and Raj 2020), as did asking people to judge the accuracy of every headline prior to making a sharing decision (Fazio 2020) or simply asking for a single accuracy judgment at the outset of the study (Pennycook et al. 2019, 2020). Indeed, encouraging individuals to think deliberately and focus on retrieving accurate information has also been shown to reduce the influence of misinformation in contexts beyond fake news—for instance, when encouraged to deliberate, fact check, and edit fictional texts with inaccurate assertions, people are less influenced by the inaccurate claims they encounter (Rapp et al. 2014).

### Emotion and engagement with fake news

Prior research has also focused in part on the roles of individuals’ emotional experiences, rather than on the use of deliberation and reason, when engaging in accuracy judgments. Different emotions have been suggested to differentially impact judgment in general, as well as perceptions of political fake news in particular. An extensive literature assesses the differential impact of specific emotions on cognition and decision-making (e.g., Appraisal-Tendency Framework; Lerner and Keltner 2001; Feelings-as-information theory; Schwarz 2011). For instance, Bodenhausen et al. (1994) found that anger elicits greater reliance upon heuristic cues in a persuasion paradigm, whereas sadness promotes an opposite, decreased reliance on heuristic cues. Literature on the relationship between emotion and gullibility has found that a negative mood state generally increases skepticism, whereas a positive mood state increases gullibility and decreases the ability to detect deception (Forgas and East 2008; Forgas 2019). Affective feelings have also been found to demonstrate a flexible influence on cognition; that is, both positive and negative emotions may improve cognitive performance, depending on the nature of the task (e.g., creative versus analytic) and processing styles available (e.g., heuristic versus systematic; see Huntsinger and Ray 2016).

More specifically within the domain of political fake news, anger has been suggested to promote politically aligned motivated belief in misinformation, whereas anxiety has been posited to increase belief in politically discordant fake news due to increased general feelings of doubt (Weeks 2015). In other words, anger may promote biased, intuitive, motivated reasoning, whereas anxiety may encourage individuals to consider opposing viewpoints (MacKuen et al. 2010) and perhaps even improve the overall quality of information seeking (Valentino et al. 2008). These hypotheses suggest that experience and use of specific emotions may elicit distinct, dissociable effects on news accuracy perception. Furthermore, evidence suggests that the illusory truth effect (i.e., believing fake news content after repeated exposure) is in some part driven by feelings of positivity cueing truth (Unkelbach et al. 2011), whereas sadness may reduce the illusory truth effect (Koch and Forgas 2012). Related research generally posits that claims are more likely to be judged as “truthful” when individuals are experiencing positive or neutral emotions, whereas negative emotions may encourage people to be more skeptical (see Brashier and Marsh 2020; Forgas 2019).

These prior assessments of the relationship between specific emotions and forming accuracy judgments are potentially also compatible with the classical reasoning account of why people fall for fake news. For instance, sad individuals may engage in analytic thinking more often and thus are more skeptical of fake news, while the opposite may be true for happy individuals (see Forgas 2019).

However, the classical reasoning account has also been conceptualized more commonly within the framework of a dual-process model of cognition, in which emotional “gut feelings” are posited to contribute to less accurate judgments and heightened belief in falsehoods. For instance, faith in intuition and one’s general feelings associated with information processing (e.g., ‘I trust my initial feelings about the facts’) have been found to be associated with belief in conspiracy theories and falsehoods in science and politics (Garrett and Weeks 2017). Furthermore, some evidence suggests that even negative emotions, generally thought to promote skepticism (Forgas 2019), can also contribute to belief in conspiracy theories, particularly when such emotions are related to the subject of the conspiracy theory (e.g., dejection-agitation; Mashuri et al. 2016). Such findings suggest that relying on existing feelings may contribute to inaccurate assessments of truth by directly increasing credulity of typically implausible content, rather than solely by reducing analytic thinking. However, prior work has yet to garner broad consensus as to the effects of experiencing or utilizing emotion per se on fake news.

### Current research

We aim to add to the current state of knowledge regarding belief in fake news in three main ways. First, little previous work has looked at the effects of experiencing specific emotions on belief in fake news. Looking at these effects will help us determine whether the potential effect(s) of emotion on fake news belief is isolated to a few specific emotions (presumably for a few idiosyncratic reasons) or whether a broader dual-process framework where emotion and reason are differentially responsible for the broad phenomenon of falling for fake news is more appropriate.

Second, much prior work on fake news has focused almost exclusively on reasoning, rather than investigating the role of emotional processing per se. In other words, prior research has treated the extent of reason and emotion as unidimensional, such that any increase in use of reason necessarily implies a decrease in use of emotion and vice-versa. In contrast, both emotion and reason may complimentarily aid in the formation of beliefs (Mercer 2010). The current study addresses this issue by separately modulating the use of reason and use of emotion. This approach, as well as the inclusion of a baseline condition in our experimental design, allows us to ask whether belief in fake news is more likely to be the result of merely failing to engage in reasoning rather than being specifically promoted by reliance on emotion. Furthermore, it allows for differentiable assessments regarding use of reason and use of emotion, rather than treating reason and emotion simply as two directions on the same continuum.

Third, prior work has been almost entirely correlational, comparing people who are predisposed to engage in more versus less reasoning. Therefore, whether a causal impact of reasoning on resistance to fake news—and/or a causal effect of emotion on susceptibility to fake news—exists remains unclear. In the current research, we address this issue by experimentally manipulating reliance on emotion versus reason when judging the veracity of news headlines.

In Study 1, we examine the association between experiencing specific emotions and believing fake news. In this study, we assess emotionality by measuring participant’s current experience of emotion prior to engaging with any news headlines (i.e., participant’s momentary “mood state”; see Rusting 1998). We examine whether heightened emotionality is associated with increased belief in fake news and decreased ability to discern between real and fake news. In Study 2, we engage in a large-scale investigation in which we separately manipulate and measure the extent to which participants utilize reason and emotion while evaluating the accuracy of news headlines. Here, we focus directly on manipulating the emotional processing (i.e., “reliance on emotion”) of individuals while judging the accuracy of news headlines (Rusting 1998). We examine whether causal evidence suggesting that inducing reliance on emotion results in greater belief in fake news exists and whether inducing reliance on reason decreases belief in fake news. We also assess whether inducing reliance on emotion or reason affects the ability to discriminate between fake and real news.

## Study 1

Study 1 investigates the association between state-based emotionality and accuracy judgments of real and fake news. In particular, we assess whether increased experience of emotion prior to viewing news headlines is associated with heightened belief in fake news headlines and decreased ability to discern between fake and real news.

## Methods

### Materials and procedure

In this exploratory study, *N* = 409 participants (227 female, *M*_age_ = 35.18) were recruited via Amazon Mechanical Turk.^1^ We did not have a sense of our expected effect size prior to this study. However, we a priori committed to our sample size (as indicated in our preregistration; https://osf.io/gm4dp/?view_only=3b3754d7086d469cb421beb4c6659556) with the goal of maximizing power within our budgetary constraints. Participants first completed demographics questions, including age, sex, and political preferences. Next, participants completed the 20-item Positive and Negative Affect Schedule scale (PANAS; Watson et al. 1988). For each item, participants were asked “To what extent do you feel [item-specific emotion] at this moment?” Likert-scale: 1 = *Very slightly or not at all*, 2 = *A little*, 3 = *Moderately*, 4 = *Quite a bit*, 5 = *Extremely*. This measure was designed to assess the current mood state of each participant.

**Fig. 1:**
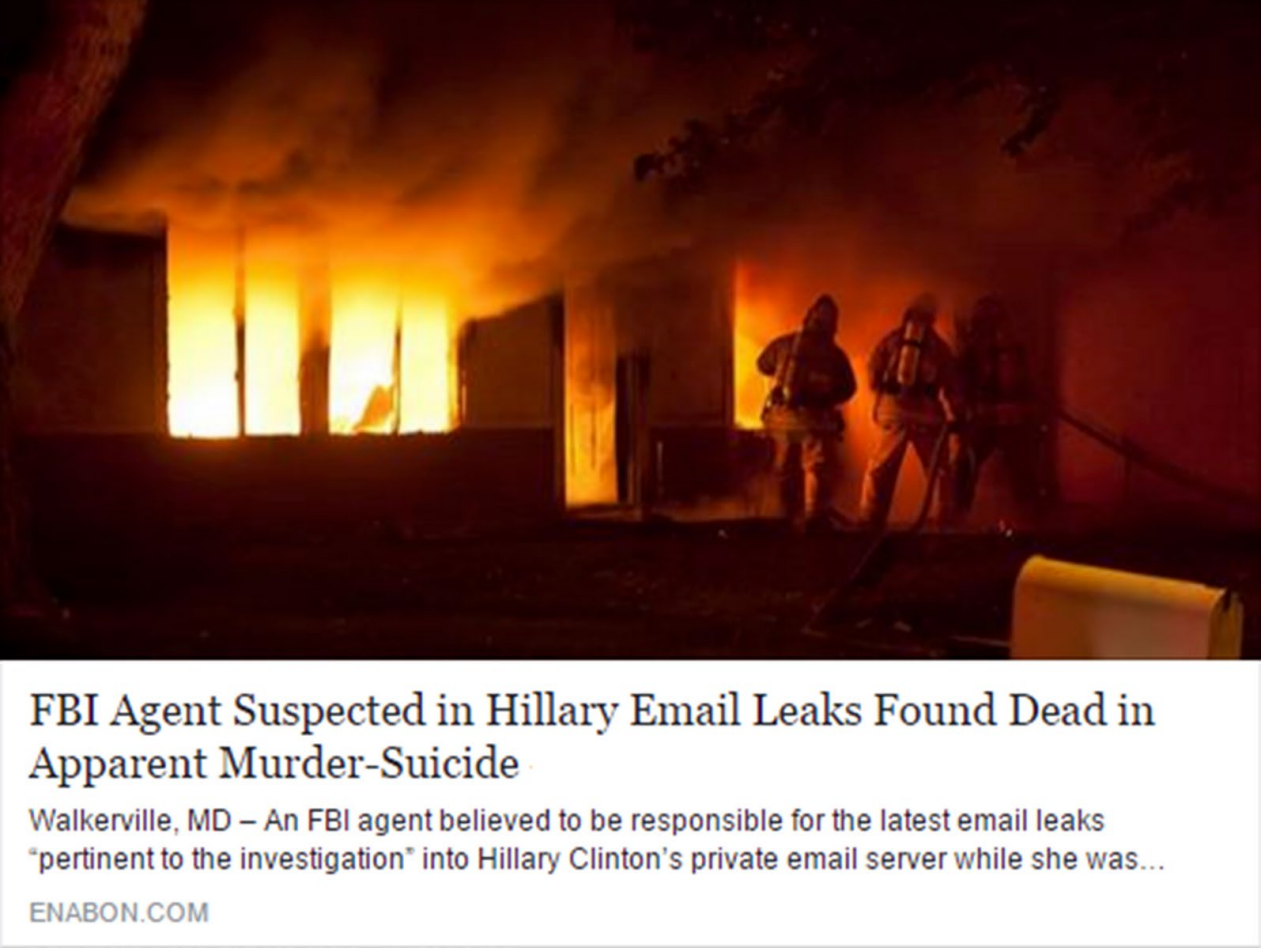
Example article with picture, headline, byline, and source. Our news items are available online (https://osf.io/gm4dp/?view_only=3b3754d7086d469cb421beb4c6659556)

After completing this measure, participants received a series of 20 actual headlines that appeared on social media, half of which were factually accurate (*real news*) and half of which were entirely untrue (*fake news*) Furthermore, half of the headlines were favorable to the Democratic Party and half were favorable to the Republican Party (based on ratings collected in a pretest, described in Pennycook and Rand 2019a). Participants in the pretest also rated the headlines on a number of other dimensions (including prior familiarity); however, they were only balanced on partisanship. These headlines were selected randomly from a larger set of 32 possible headlines—again half real, half fake, and half Democrat-favorable, and half Republican-favorable. All fake news headlines were taken from Snopes.com, a well-known fact-checking website. Real news headlines were selected from mainstream news sources (e.g., NPR, The Washington Post) and selected to be roughly contemporary to the fake news headlines. The headlines were presented in the format of a Facebook post—namely, with a picture accompanied by a headline, byline, and a source (see Fig. 1). For each headline, participants were asked: “To the best of your knowledge, how accurate is the claim in the above headline” using a 4-point Likert-scale: 1 = Not at all accurate, 2 = Not very accurate, 3 = Somewhat accurate, 4 = Very accurate.

## Results and discussion

### Across emotions, greater emotionality predicts increased belief in fake news and decreased truth discernment

In our first analysis, we assessed the relationship between emotionality (i.e., momentary mood state of experiencing a particular emotion) and perceived accuracy of real and fake news. We used the R packages *lme4* (Bates et al. 2015), *lmerTest* (Kuznetsova et al. 2017), and *arm* (Gelman and Su 2018) to perform linear mixed-effects analyses of the relationship between perceived accuracy, specific emotions measured by the PANAS, and type of news headline (fake, real). A mixed-effects model allows us to account for the interdependency between observations due to by-participant and by-item variation. We entered the PANAS score for the item of interest, type of news headline, and an interaction between the two terms into the model as fixed effects. We had intercepts for headline items and participants, as well as by-item random slopes for the effect of the PANAS emotion-item rating and by-participant random slopes for the effect of type of news headline, for random effects. The reference level for type of news headline was “fake.” Since 20 emotions were assessed by the PANAS, we performed 20 linear mixed-effects analyses. To further demonstrate the generalizability of our results across emotions, we also performed two additional linear mixed-effects analyses with aggregated PANAS scores for negative and positive emotions, which were calculated via a varimax rotation on a two-factor analysis of the 20 PANAS items. The beta coefficients for the interaction between emotion and news type are reported as “Discernment” (i.e., the difference between real and fake news, with a larger coefficient indicating higher overall accuracy in media truth discernment), and the betas for real news were calculated via joint significance tests (i.e., F-tests of overall significance). Our results are summarized in Table 1.^2^

**Table 1:** Results of linear mixed-effects analyses for each emotion measured by the PANAS scale

	Enthusiastic	Interested	Determined	Excited	Inspired	Alert	Active
Fake	0.13^c^	0.04	0.07^b^	0.15^c^	0.15^c^	0.05^a^	0.10^c^
Real	0.01	0.03	0.01	0.002	0.001	0.05^a^	0.01
Discernment	− 0.11^c^	− 0.02	− 0.06	− 0.14^c^	− 0.15^c^	− 0.01	− 0.09^b^

	Strong	Proud	Attentive	Scared	Afraid	Upset	Distressed
Fake	0.10^c^	0.11^c^	0.01	0.15^c^	0.13^c^	0.11^c^	0.12^c^
Real	− 0.01	− 0.03	0.04^a^	− 0.02	− 0.02	0.003	0.003
Discernment	− 0.10^b^	− 0.14^c^	0.03	− 0.17^c^	− 0.15^c^	− 0.11^c^	− 0.11^c^

	Jittery	Nervous	Ashamed	Hostile	Guilty	Irritable	Positive	Negative
Fake	0.11^c^	0.10^c^	0.12^c^	0.15^c^	0.09^c^	0.11^c^	0.13^c^	0.17^c^
Real	− 0.01	− 0.01	− 0.03	− 0.01	− 0.02	− 0.001	0.01	− 0.02
Discernment	− 0.13^c^	− 0.11^b^	− 0.15^c^	− 0.16^c^	− 0.11^b^	− 0.11^b^	− 0.12^c^	− 0.18^c^

Fixed effects in model include PANAS item score, type of news headline, and interaction between PANAS score and type of news headline. Random effects include random intercepts for headline items and participants and by-item random slopes for PANAS scores and by-participant random slopes for type of news headline effects

^a^*p* < 0.05

^b^*p* < 0.01

^c^*p* < 0.001

Overall, our results indicate that, for nearly every emotion evaluated by the PANAS scale,^3^ increased emotionality is associated with increased belief in fake news. Furthermore, we also find that nearly every emotion also has a significant interaction with type of news headline, such that greater emotionality also predicts decreased discernment between real and fake news. Indeed, the only emotions for which we do not see these effects are “interested,” “alert,” “determined,” and “attentive,” which arguably are all more closely associated with analytic thinking rather than emotionality per se; however, although we do not find significant relationships between these emotions and belief in fake news or discernment, we also do not provide evidence that such relationships do not exist. Our results also suggest that the relationship between emotion and news accuracy judgments appear to be specific to fake news; that is, for every emotion except “attentive” and “alert,” no significant relationship exists with real news belief. Our key findings are also robust when controlling for headline familiarity (see Additional file 1, which contains descriptive statistics and additional analyses).

We not only find statistically significant associations between experiencing emotion and believing fake news but also observe rather substantial effect sizes. Our mixed-effects model indicates that belief in fake news (relative to the scale minimum value of 1) is nearly twice as high for participants with the highest aggregated positive and negative emotion scores (accuracy ratings of 0.96 and 1.45 above scale minimum, respectively) compared to participants with the lowest aggregated positive and negative emotion scores (accuracy ratings of 0.34 and 0.50 above scale minimum, respectively). Therefore, although even participants who experience high emotion are still, on average, able to discern between fake and true news, we observe notable increases in belief in fake news as emotionality increases.

**Fig. 2:**
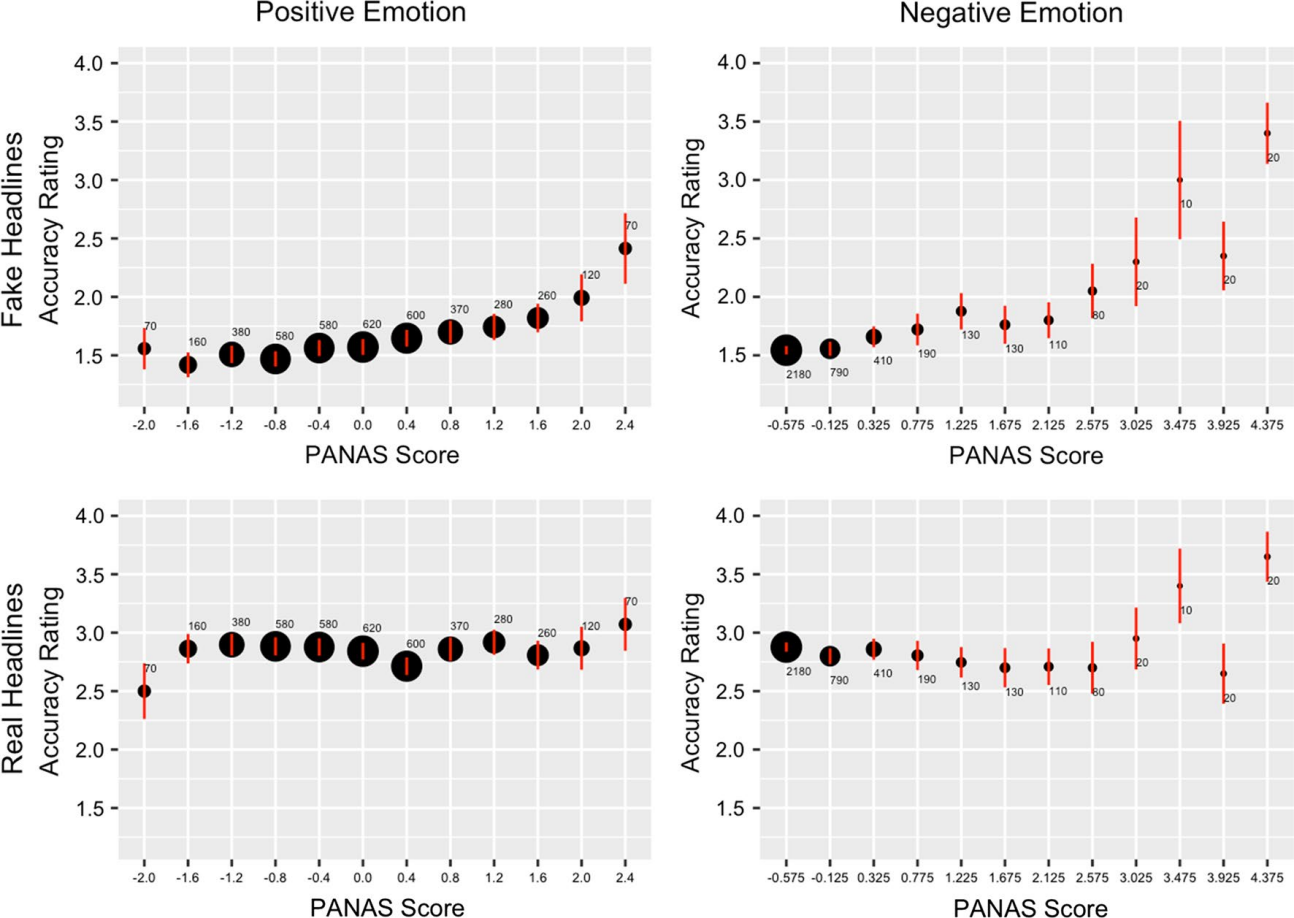
Plotting reported news headline accuracy as a function of aggregated positive or negative PANAS score shows a positive relationship between both positive and negative emotion and belief in fake news. This relationship is not as evident for belief in real news. Dot size is proportional to the number of observations (i.e., a specific participant viewing a specific headline). Error bars, mean ± 95% confidence intervals

As shown by most of our 20 previous linear mixed-effects models, both positive and negative emotion are associated with higher accuracy ratings for fake headlines (Fig. 2), and this relationship does not exist as clearly for real headlines.

#### Interactions with headline political concordance

Some prior work has argued that an interaction may exist between specific types of emotions and political concordance of news when assessing belief in fake news (e.g., Weeks 2015). Therefore, we next performed multiple linear mixed-effects analyses of the relationship between specific emotions, type of news headline, participant’s partisanship (z-scored; continuous Democrat vs. Republican), and headline political concordance (z-scored; concordant (participant and headline partisanship align), discordant (participant and headline partisanship oppose)), allowing for interactions between all items. Our maximal linear mixed model failed to converge, so we followed the guidelines for how to achieve convergence in Brauer and Curtin (2018), and removed the by-unit random slopes for within-unit predictors and lower-order interactions, leaving the by-unit random slopes for the highest order interactions (see also: Barr 2013). This left us with by-item random slopes for the interaction between PANAS emotion, concordance, and political party and by-participant random slopes for the interaction between type of headline and concordance. We again assessed how each emotion was associated with belief in fake news and real news, as well as the interaction between news type and emotion. Furthermore, we also assessed the interaction between emotion and concordance for fake news, as well as the three-way interaction among news type, emotion, and political concordance (reported as “Discernment × Concordant”). Our key results are summarized in Table 2.

**Table 2:** Results of linear mixed-effects analyses for each emotion measured by the PANAS scale, plus interaction with headline political concordance

	Enthusiastic	Interested	Determined	Excited	Inspired	Alert	Active
Fake	0.13^c^	0.04^a^	0.05^a^	0.14^c^	0.14^c^	0.05^b^	0.10^c^
Real	0.02	0.03	0.02	0.01	0.004	0.06^b^	0.01
Discernment	− 0.10^c^	− 0.02	− 0.04	− 0.14^c^	− 0.14^c^	0.002	− 0.09^c^
Fake × concordant	− 0.01	0.01	0.01	− 0.01	− 0.004	− 0.004	− 0.01
Discernment × concordant	− 0.001	− 0.01	0.005	0.003	− 0.01	0.01	0.01

	Strong	Proud	Attentive	Scared	Afraid	Upset	Distressed
Fake	0.09^c^	0.10^c^	0.01	0.15^c^	0.13^c^	0.11^c^	0.12^c^
Real	− 0.003	− 0.03	0.04^a^	− 0.02	− 0.03	0.001	− 0.01
Discernment	− 0.10^c^	− 0.13^c^	0.03	− 0.17^c^	− 0.16^c^	− 0.11^c^	− 0.12^c^
Fake × concordant	− 0.002	− 0.03^a^	− 0.02	− 0.04^b^	− 0.03^a^	− 0.03^a^	− 0.03^b^
Discernment × concordant	0.02	0.03	0.03	0.01	− 0.01	0.01	0.0003

	Jittery	Nervous	Ashamed	Guilty	Irritable	Hostile	Positive	Negative
Fake	0.12^c^	0.10^c^	0.12^c^	0.09^c^	0.11^c^	0.15^c^	0.13^c^	0.17^c^
Real	− 0.02	− 0.01	− 0.02	− 0.02	− 0.1	− 0.01	0.02	− 0.02
Discernment	− 0.13^c^	− 0.11^c^	− 0.14^c^	− 0.11^c^	− 0.12^c^	− 0.16^c^	− 0.11^c^	− 0.19^c^
Fake × concordant	− 0.02	− 0.03^a^	− 0.03^b^	− 0.03^a^	− 0.03^b^	− 0.03^a^	− 0.01	− 0.04^b^
Discernment × concordant	0.02	− 0.004	0.001	0.001	0.01	0.01	0.01	0.01

Fixed effects in model include PANAS item score, type of news headline, partisanship, and political concordance. Random effects in model include by-item random slopes for the interaction between PANAS item score, concordance, and political party, and by-participant random slopes for the interaction between type of headline and concordance

^a^*p* < 0.05

^b^*p* < 0.01

^c^*p* < 0.001

As with our prior models, we again find that for nearly all of the emotions assessed by the PANAS, greater emotionality is associated with heightened belief in fake news and decreased discernment between real and fake news. Emotion also appears to selectively affect fake news judgment and is unrelated to belief in real news. Looking at the interaction between emotion and concordance, our results are less consistent: some emotions significantly interact with concordance, though these coefficients are relatively small compared to the interaction with type of news. Our results also suggest that a significant interaction exists between negative emotion and concordance but not between positive emotion and concordance, indicating some specificity of effects of emotion on belief in fake news. However, no differences are observed between emotions hypothesized to have differentiable effects on belief in fake news. For example, emotions such as “hostile” and “nervous” similarly interact with political concordance. This finding is in contrast with those of Weeks (2015), who suggests that anger selectively heightens belief in politically concordant fake news, while anxiety increases belief in politically discordant fake news. Rather, our results instead tentatively suggest that emotion in general heightens belief in fake news and that different emotions do not necessarily interact with political concordance in a meaningful way. Furthermore, across all emotions, no significant three-way interactions were observed among news type, emotion, and political concordance, and therefore, we do not find evidence suggesting that political concordance interacts with the relationship between emotion and discernment.

A potential limitation of Study 1 is that our results could be in partly driven by floor effects, as most participants self-reported experiencing a relatively low level of emotion. However, the average mean score across all twenty individual emotions (*M* = 2.19) and the average median score across all twenty emotions (*M* = 1.95) were relatively similar, and both were still well above the lowest end of the PANAS scale. To verify that our results are not being driven primarily by floor effects, we also analyzed the relationships between aggregated positive and negative emotion and news accuracy ratings while only including participants who had above the median scores for positive and negative emotion, respectively. Looking at the relationship between aggregated positive emotion and belief in news headlines for only participants with above-median positive emotion, we still find that greater positive emotion relates to increased belief in fake headlines (*b* = 0.23, SE = 0.06, *t*(135.44) = 3.93, *p* < 0.001), and that greater positive emotion results in decreased discernment between real and fake news (*b* = − 0.17, SE = 0.07, *t*(111.60) = − 2.34, *p* = 0.021. We again do not find that greater positive emotion relates to increased belief in real headlines (*p* = 0.239). Similarly, looking at the relationship between aggregated negative emotion and belief in news headlines for participants with above-median negative emotion, we again find that greater negative emotion relates to increased belief in fake headlines (*b* = 0.19, SE = 0.03, *t*(117.46) = 5.60, *p* < 0.001), and that greater negative emotion results in decreased discernment between real and fake news (*b* = − 0.20, SE = 0.05, *t*(105.60) = − 4.24, *p* < 0.001). We once again do not find that greater negative emotion relates to increased belief in fake headlines (*p* = 0.887).

Another potential concern with Study 1 is that participants with higher PANAS scores are simply less attentive, and these inattentive participants are those performing worse on discriminating between real and fake news. However, this alternative explanation does not account for our findings that certain emotions (e.g., interested, alert, attentive) are not associated with decreased discernment between real and fake news, which demonstrate that our correlational findings are specific to a distinct set of emotions assessed by the PANAS, thus alleviating some concerns of floor effects driving our results.

Taken together, the results from Study 1 suggest that emotion in general, regardless of the specific type of emotion, predicts increased belief in fake news. Furthermore, nearly every type of emotion measured by the PANAS also appears to have a significant interaction with type of news, indicating an effect of emotion on differentiating real from fake news. Therefore, in Study 2, we causally assess the role of emotion in fake news perception using a dual-process framework—in which reliance on emotion in general is contrasted with reliance on reason—rather than by differentially assessing various roles of experiencing specific emotions.

## Study 2

Study 2 expands on the findings of Study 1 in several ways. First, Study 1 found that experienced emotion, regardless of the specific type of emotion, was associated with increased belief in fake news, as well as decreased ability to differentiate between real and fake news. To explain this association, we hypothesized that individuals who experienced greater emotionality also relied on emotion to a greater extent when making accuracy judgments of news headlines (otherwise, why increased emotionality should impact decision-making is not clear). Therefore, in Study 2, we directly manipulate the way that individuals engage in emotional processing while evaluating the veracity of news headlines. We manipulate the extent to which individuals rely on emotion (in general^4^) or reason when judging the accuracy of news headlines. We investigate whether reliance on emotion versus reason causally affects judgments of fake news, as well as the ability to discern between real and fake news.

## Methods

### Materials and procedure

Our results from Study 1 suggest that heightened emotion in general is predictive of increased belief in fake news. To further assess the relationship between emotion and fake news belief, Study 2 analyzes a total of four experiments that shared a virtually identical experimental design in which reliance on reason versus emotion was experimentally manipulated using an induction prompt from Levine et al. (2018). The general procedure across all four experiments was as follows. Participants were randomly assigned to one of three conditions: a reason induction (“Many people believe that reason leads to good decision-making. When we use logic, rather than feelings, we make rationally satisfying decisions. Please assess the news headlines by relying on reason, rather than emotion.”), an emotion induction (“Many people believe that emotion leads to good decision-making. When we use feelings, rather than logic, we make emotionally satisfying decisions. Please assess the news headlines by relying on emotion, rather than reason.”), or a control induction (with the exception of experiment 1, which had no control condition (see Table 3); participants in all three conditions first read “You will be presented with a series of actual news headlines from 2017–2018. We are interested in your opinion about whether the headlines are accurate or not.”). After reading the induction prompt, participants receive a series of actual headlines that appeared on social media, some of which were factually accurate (*real news*), some of which were entirely untrue (*fake news*), some of which were favorable to the Democratic party, and some of which were favorable to the Republican party (based on ratings collected in a pretest, described in Pennycook and Rand 2019a). Fake and real news headlines were selected via a process identical to that described in Study 1. Our news items are available online (https://osf.io/gm4dp/?view_only=3b3754d7086d469cb421beb4c6659556). For each headline, real or fake, perceived accuracy was assessed. Participants were asked: “How accurate is the claim in the above headline?” Likert-scale: 1 = *Definitely false,* 2 = *Probably false,* 3 = *Possibly false*, 4 = *Possibly true,* 5 = *Probably true*, 6 = *Definitely true*. The specific number of fake, real, pro-Democrat, and pro-Republican headlines each participant viewed varied by experiment (see *News headlines* section of Table 3).

**Table 3:** Description of participants, methods, and measures for each experiment

	Experiment 1	Experiment 2	Experiment 3	Experiment 4
Participants	472 from Amazon Mechanical Turk (*M*_age_ = 35.12, 243 female)	1108 from Amazon Mechanical Turk (*M*_age_ = 35.19, 618 female)	1129 from Amazon Mechanical Turk (*M*_age_ = 34.40, 645 female)	1175 from Lucid^a^ (*M*_age_ = 45.46, 606 female)
Conditions	Emotion induction, reason induction	Emotion induction, reason induction, control	Emotion induction, reason induction, control	Emotion induction, reason induction, control
News headlines	6 fake headlines (half democrat-consistent, half Republican-consistent)	6 fake, 6 real headlines (half democrat-consistent, half Republican-consistent)	5 fake, 5 real headlines (all politically concordant based on force-choice Trump versus Clinton question)	6 fake, 6 real headlines (half Democrat-consistent, half Republican-consistent)
Scale questions on use of reason/emotion (Likert: 1–5)	Not included	Included	Included	Included
Participant Inclusion Criteria	Restricted to United States; 90% HIT Approval Rate	Restricted to United States; 90% HIT Approval Rate	Restricted to United States; 90% HIT Approval Rate	Typical Lucid Representative Sample

Lucid, an online convenience sampling platform comparable to Mechanical Turk, is purported to have a larger pool of subjects than MTurk, less professionalized subjects, and subjects more similar to US benchmarks regarding demographic, political, and psychological profiles (see Coppock and McClellan 2019)

After rating the headlines, participants completed various post-experimental questionnaires. Most relevant for the current paper, participants were asked if they preferred that Donald Trump or Hillary Clinton was the President of the United States.^5^ Pro-Democratic headlines rated by Clinton supporters and Pro-Republican headlines rated by Trump supporters were classified as politically concordant headlines, whereas Pro-Republican headlines rated by Clinton supporters and Pro-democratic headlines rated by Trump supporters were classified as politically discordant headlines.

Participants also completed a free-response manipulation check in which they were asked the question “At the beginning of the survey, you were asked to respond using your__” with words related to “emotion” or “intuition” being scored as accurate for the emotion induction condition and words relating to “reason” or “logic” being scored as accurate for the reason induction condition. Participants were also asked “At the beginning of the survey, you were asked to respond using your:” 1 = *Emotion*, 2 = *Reason*.

Participants in experiments 2 through 4 further completed several questions asking about the extent to which they used reason or emotion. Participants were directed to “Please indicate the extent to which you used emotion/feelings when judging the accuracy of the news headlines” and “Please indicate the extent to which you used reason/logic when judging the accuracy of the news headlines” according to the following Likert scale: 1 = *None at all*, 2 = *A little*, 3 = *A moderate amount*, 4 = *A lot*, 5 = *A great deal*.

Participants also completed several other measures (a shortened version of the actively open-minded thinking scale; Stanovich and West 2007; a reworded version of the original Cognitive Reflection Test, a measure of analytic thinking; CRT; Frederick 2005; Shenhav et al. 2012; and a four-item non-numeric CRT; Thomson and Oppenheimer 2016) and standard demographics (e.g., age, sex, education), but we do not analyze those responses here. These further measures were included for exploratory purposes and are not analyzed or discussed here. However, all measures are included in our openly available aggregated data (see https://osf.io/gm4dp/?view_only=3b3754d7086d469cb421beb4c6659556). Furthermore, see Table 3 for further details on each experiment’s participants, design, and procedures.

We completed preregistrations of sample size, experimental design, and analyses for each experiment (available online https://osf.io/gm4dp/?view_only=3b3754d7086d469cb421beb4c6659556). Note that, across all four preregistrations, we predicted that analytic thinking should improve discernment between real and fake news.

We again did not have a sense of our expected effect sizes prior to running these studies. However, we a priori committed to our sample size (as indicated in our preregistrations) with the goal of maximizing power within our budgetary constraints. Additionally, our sample sizes are quite large relative to typical sample sizes in this field.

We soon recognized that the subject-level analysis approach proposed in all the preregistrations—calculating each subject’s average accuracy rating for each type of headline and performing an ANOVA predicting these subject-level averages based on condition and headline type—is problematic and may introduce bias (Judd et al. 2012). Thus, we do not follow our preregistered analyses and instead follow the guidelines of Judd et al. by conducting rating-level analyses using linear mixed-effects models with crossed random effects for subject and headline.

Furthermore, since all four experiments had essentially identical designs (in particular, manipulated reliance on emotion and reason, and asked for judgments of headline accuracy), we aggregate the data from each experiment and nest the subject within experiment in our random effects. Thus, none of the analyses reported in this paper were preregistered; however, we note that our decision to aggregate the four studies was made after we decided that we would not run any additional studies, and thus, our stopping criterion was not based on the outcome of the aggregate analysis. We aggregated our data across all four studies for several reasons. First, this substantially improved our statistical power for assessing the relative roles of relying on emotion and relying on reason in the formation of news headline accuracy judgments. Second, by combining across multiple studies, we could examine whether the effects of reliance on emotion or reliance on reason on media truth judgments were existent or consistent across a range of slightly different assessments, or if such relationships only appear in particular individual experiments.

## Results and discussion

### Correlational results

#### Greater reliance on reason relative to emotion predicts greater truth discernment

Before assessing the results of our causal manipulation, we examined the correlational relationship between self-reported use of reason, use of emotion, and headline accuracy ratings from the control conditions across experiments 2 through 4 (*N* = 1089). We start by investigating the *relative* use of reason versus emotion, and then (as argued above), we treat reason and emotion as separate continua and investigate their unique roles in fake/real news belief.

We first calculated relative use of reason as a difference score of self-reported use of reason minus self-reported use of emotion. We then performed a linear mixed-effects analysis of the relationship between perceived accuracy, relative use of reason versus emotion, and type of news headline (fake, real). Experiment (i.e., “study”) was also included in the model as a categorical covariate. We entered the relative use of reason, type of news headline, an interaction between the two terms, and study into the model as fixed effects. We had intercepts for headline items and participants nested by study, as well as by-item random slopes for the effect of relative use of reason and by-nested participant random slopes for the effect of type of news headline as random effects. The reference level for type of news headline was “fake.” Consistent with the classical account, we found that participants who self-reported greater relative use of reason rated fake news as less accurate, *b* = − 0.17, SE = 0.02, *t*(67.14) = − 7.34, *p* < 0.001. A significant interaction existed between relative use of reason and type of news headline, *b* = 0.20, SE = 0.03, *t*(48.66) = 6.65, *p* < 0.001, such that no effect of relative use of reason on perception of real headlines, *b* = 0.02, *F*(1, 52.94) = 1.29, *p* = 0.260, was observed. Thus, we found that participants who self-reported greater relative use of reason exhibited better discernment between news types. All study dummies were nonsignificant (*p* > 0.05). These findings are robust in the control for headline familiarity (see Additional file 1).

#### Unique relationships with use of emotion versus reason

We next ran a linear mixed-effects analysis similar to the aforementioned model, except replacing relative use of reason with either self-reported use of emotion or self-reported use of reason. When we considered use of emotion, we found that participants who reported greater use of emotion rated fake news headlines as more accurate, *b* = 0.26, SE = 0.03, *t*(48.14) = 8.08, *p* < 0.001. We also found a significant interaction between use of emotion and type of news headline, *b* = − 0.22, SE = 0.04, *t*(38.33) = − 5.24, *p* < 0.001, such that there was no effect of use of emotion on perceptions of real headlines, *b* = 0.04, *F*(1, 40.39) = 2.29, *p* = 0.138. Study dummies were again nonsignificant (*p* > 0.05).

**Fig. 3:**
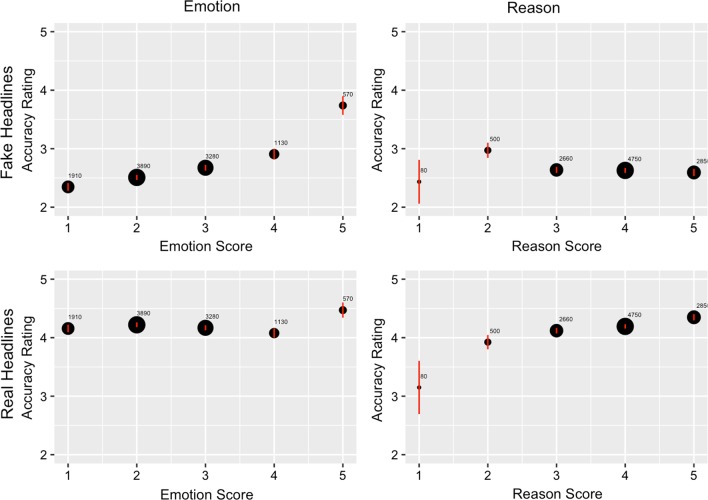
Plotting reported news headline accuracy as a function of use of emotion or use of reason shows a positive relationship between emotion and belief in fake news, and a positive association between reason and belief in real news. Dot size is proportional to the number of observations (i.e., a specific participant viewing a specific headline). Error bars, mean ± 95% confidence intervals

Conversely, when we considered use of reason, we found no significant relationship between use of reason and accuracy ratings of fake news, *p* > 0.05. However, a significant interaction was observed between use of reason and type of news, *b* = 0.17, SE = 0.04, *t*(78.82) = 4.27, *p* < 0.001, because use of reason was positively associated with perceived accuracy of real headlines, *b* = 0.22, *F*(1, 77.23) = 20.94, *p* < 0.001. Study dummies were again nonsignificant (*p* > 0.05). This evidence suggests that use of emotion may be uniquely linked to belief in false content whereas use of reason is uniquely linked to belief in true content. Figure 3 visually summarizes the results of our analyses: use of emotion is positively associated with belief in fake news but not real news, and use of reason is positively associated with belief in real news but is unrelated to belief in fake news. These findings, as well as our use of emotion findings, both remain largely consistent when we controlled for headline familiarity (see Additional file 1).

#### Interactions with participant partisanship and headline political concordance

We then performed a linear mixed-effects analysis of the relationship between relative use of reason, type of news headline, participant’s partisanship (Clinton supporter, Trump supporter), and headline political concordance (concordant, discordant), allowing for interactions between all terms. Study was added as a covariate, without interactions. Our maximal linear mixed model failed to converge, so we followed the guidelines for how to achieve convergence in Brauer and Curtin (2018) and removed the by-unit random slopes for within-unit predictors and lower-order interactions, while leaving the by-unit random slopes for the highest order interactions (also see Barr 2013). As a result, our random effects included intercepts for headline items and participants nested by study; by-item random slopes for the three-way interaction among relative use of reason, concordance, and partisanship; and by-nested participant random slopes for the interaction between type of headline and concordance. The reference levels were “fake” for news type, “Clinton” for partisanship, and “discordant” for concordance. As in our model without partisanship and concordance, we found that relative use of reason was negatively associated with perceived accuracy of fake stories (*p* < 0.001) and had a significant interaction with type of headline (*p* < 0.001), such that no relationship was observed between relative use of reason and real news perception, *b* = 0.01, *F*(1, 114.61) = 0.12, *p* = 0.730. We found no effect of study (*p* > 0.05).

Our model also suggested a significant interaction between relative use of reason and concordance, *b* = 0.11, SE = 0.02, *t*(10,240) = 4.41, *p* < 0.001. The motivated account of fake news would predict that higher relative reasoners perceive concordant fake news as more accurate as compared to lower relative reasoners. However, we found the opposite: for concordant fake news headlines, relative use of reason was associated with decreased accuracy ratings, *b* = − 0.09, *F*(1, 609.63) = 9.72, *p* = 0.002. Both accounts would predict higher relative reasoners to perceive concordant real news as more accurate. We found that relative use of reason was nominally positively associated with accuracy ratings of concordant real news headlines, *b* = 0.05, *F*(1, 600.57) = 3.08, *p* = 0.080, though this relationship was not statistically significant.

Our model also revealed a three-way interaction among relative use of reason, type of news, and partisanship, *b* = − 0.04, SE = 0.02, *t*(5,200) = − 2.58, *p* = 0.010. For both Clinton and Trump supporters, relative use of reason was negatively associated with perceived accuracy of fake headlines (*b* = − 0.20 for both). The relationship between relative use of reason and perceived accuracy of real headlines, however, differed slightly based on partisanship: for Clinton supporters, the relationship was (barely) positive, *b* = 0.01, whereas for Trump supporters the relationship was somewhat negative, *b* = − 0.04. However, neither of the latter two effects were themselves significant (*p* > 0.1 for both); thus, we do not think that this three-way interaction is particularly meaningful.

### Experimental manipulation results

#### Manipulation check of causal manipulation

A brief manipulation check reveals that, across all four experiments, participants reported greatest use of emotion in the emotion condition (*M* = 3.47), followed by in the control condition (*M* = 2.50) and the reason condition (*M* = 2.06), *F*(2, 3386) = 479.80, *p* < 0.001. Similarly, participants reported greatest use of reason in the reason condition (*M* = 4.14), followed by in the control condition (*M* = 3.90) and the emotion condition (*M* = 2.91), *F*(2, 3395) = 479.20, *p* < 0.001. Follow-up pairwise Tukey tests revealed significant differences between all conditions for both use of emotion and reason, *p* < 0.001.

Participants also reported greatest relative use of reason in the reason condition (*M* = 2.08), followed by the control condition (*M* = 1.41), and finally the emotion condition (*M* = − 0.56), *F*(2, 3372) = 748.60, *p* < 0.001. These results suggest that (1) participants used relatively more emotion than reason in the emotion condition, (2) participants used relatively more reason than emotion in the reason and control conditions (based on self-report), and (3) the self-reported relative use of reason in the control condition was more similar to that of the reason condition than the emotion condition—suggesting that the manipulation was more successful at shifting people who typically rely on reason towards emotion than vice versa.

We also assessed how adherence to our manipulations was associated with headline accuracy ratings across conditions (see Additional file 1).

#### Manipulation effect on news accuracy perceptions

We next examined whether there was a condition effect on the perceived accuracy of fake and real news across all four experiments. We performed a linear mixed-effects analysis of the relationship between perceived news accuracy, experimental condition (emotion, control, reason), and type of news headline. We entered condition and type of news headline as fixed effects, with an interaction term. We also added study as a covariate. We included intercepts for headline items and participants nested by study, as well as by-item random slopes for condition and by-nested participant random slopes for type of news headline, as random effects. The reference level for condition was “emotion” and the reference level for type of news headline was “fake.” The results of this analysis are shown in Table 4,^6^ (with “study” variables omitted, no effect of study was observed; all *p* > 0.05).

**Table 4:** Results of linear mixed-effects analysis of accuracy by condition and type of news article

	beta	SE	*df*	*|t|*	*p*
Intercept	2.32	1.69	0.0002	1.37	.999
Control (condition)	− 0.12	0.04	140.20	− 3.01	.003
Reason (condition)	− 0.09	0.04	102.60	− 2.23	.028
Real (headline truth)	1.21	0.14	38.00	8.36	< 0.001
Control: real	0.10	0.05	75.99	2.01	.048
Reason: real	0.11	0.05	61.77	2.20	.031

Fixed effects in model include experimental condition and type of news headline, plus their interaction. Random effects in model include random intercepts for headline items and participants nested by study, as well as by-item random slopes for condition and by-nested participant random slopes for type of news headline

A joint significance test revealed a significant effect of condition on fake news accuracy judgments, *F*(2, 186.54) = 4.72, *p* = 0.010.^7^ From our model, we see that fake news headlines were reported as significantly more accurate in the emotion condition as compared to the control condition (*p* = 0.003) and the reason condition (*p* = 0.028), respectively.

With respect to the magnitude of our condition effect on belief in fake news, we observe approximately a 10% increase in belief from our control condition (1.20 above scale minimum) to our emotion condition (1.32 above scale minimum) according to our mixed-effects model. While participants are still largely able to discern between real and fake news even in our emotion condition, this effect size suggests that belief in fake news was still meaningfully increased by the emotion induction.

**Fig. 4:**
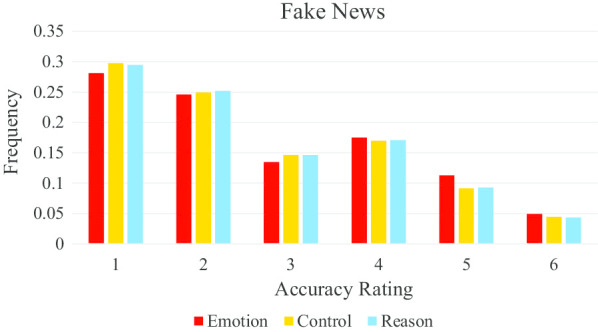
Higher accuracy ratings were more frequently given to fake news headlines in the emotion condition compared to the control and reason conditions

Figure 4 shows that participants in the emotion condition more frequently assigned higher accuracy ratings to fake stories, whereas participants in the control and reason conditions more frequently assigned low accuracy ratings to fake stories.

**Fig. 5:**
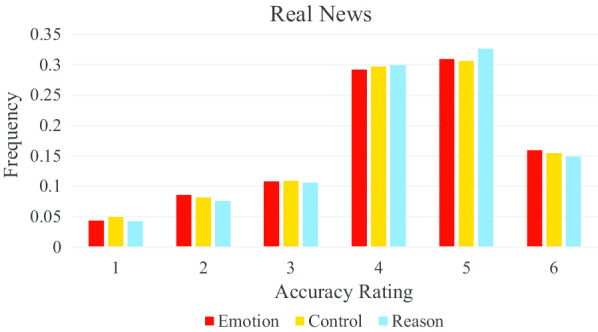
All three conditions produce similar accuracy ratings of real news stories

In contrast, a joint significance test of condition on real news accuracy perception did not show a significant effect, *F*(2, 114.42) = 1.18, *p* = 0.312. That is, no effect was observed of thinking mode on real news accuracy perception (see Fig. 5).

We next performed a joint significance test of the interaction between condition and news type. This revealed a marginally significant interaction, *F*(2, 112.60) = 2.75, *p* = 0.069. The coefficients of our model show that media truth discernment, as indicated by the interaction between condition and news type, is significantly greater in the control condition than in the emotion condition (*p* = 0.048) and also significantly greater in the reason condition than in the emotion condition (*p* = 0.031) but did not significantly differ between the reason condition and the control condition (*p* = 0.821), hence, the larger *p* value for the joint significance test. Therefore, only a marginal effect was noted of condition on media truth discernment, such that discernment is worst in the emotion condition and comparatively better in both the control and reason conditions. Given that discernment is greater in the control condition than in the emotion condition, as well as greater in the reason condition than in the emotion condition, our results tentatively suggest that emotional thinking may hinder the ability to discern fake from real news. However, our results of an overall condition effect on truth discernment are not statistically significant, suggesting that manipulating emotion versus reason may not influence discernment overall compared to a control condition.

#### Interactions with participant partisanship and headline concordance

We next performed a linear mixed-effects analysis including partisanship and political concordance. Our maximal linear mixed model failed to converge, so we followed the guidelines for how to achieve convergence in Brauer and Curtin (2018). Ultimately, the only model that would converge was a model with random intercepts but without random slopes, which does inflate Type I error rate (Barr 2013). Our fixed effects included condition, real, concordance, and partisanship, allowing for all interactions. Study was included as a covariate without interactions. Our random effects included intercepts for headline items and participants nested by study. The reference levels were “fake” for news type, “Clinton” for partisanship, and “discordant” for concordance.

According to the motivated account, an interaction should exist between condition and concordance, such that fake concordant headlines have higher perceived accuracy in the reason condition than the emotion condition, and fake discordant headlines have lower perceived accuracy in the reason condition than the emotion condition. However, a joint significance test of the interaction between condition and concordance revealed a nonsignificant interaction, *F*(2, 39,081.07) = 1.09, *p* = 0.335. A joint significant test of the three-way interaction among condition, concordance, and type of news headline also yielded nonsignificant results, *F*(2, 36,302.32) = 0.45, *p* = 0.636.

However, joint significance was observed for the three-way interaction among condition, type of news, and partisanship, *F*(2, 36,946.68) = 4.24, *p* = 0.014. Yet, follow-up analyses did not yield any significant differences in discernment across conditions for Clinton supporters or Trump supporters. For Clinton supporters, discernment in the emotion condition was nominally (though nonsignificantly) lower (*M* = 1.73) than discernment in either the control condition (*M* = 1.86) or reason condition (*M* = 1.81). Interestingly, for Trump supporters, discernment scores in the emotion (*M* = 1.11) and control (*M* = 1.12) conditions were nominally lower than in the reason condition (*M* = 1.26). Notably, none of these differences were statistically significant, perhaps due to the reduction in sample size—and thus power—arising from sub-setting for partisanship. Nonetheless, we found it potentially interesting that in the control condition, Clinton supporters exhibit media truth discernment capabilities more similar to the reason condition, whereas Trump supporters exhibit media truth discernment more similar to the emotion condition.

A joint significant test also revealed a significant three-way interaction among condition, concordance, and partisanship, *F*(2, 39,042.94) = 5.52, *p* = 0.004. This three-way interaction was such that Clinton supporters nominally, though not significantly, perceived concordant fake headlines as most accurate in the emotion condition (*M* = 2.88) and as less accurate in both the control and reason conditions (*M*’s = 2.76), while Trump supporters perceived concordant fake headlines as nominally most accurate in both the emotion (*M* = 3.16) and reason (*M* = 3.15) conditions, and as least accurate in the control condition (*M* = 3.05). Interestingly, this pattern also emerged in Clinton supporters’ perceptions of discordant fake headlines, with higher accuracy perceptions in the emotion and reason conditions (*M*’s = 2.21) than in the control condition (*M* = 2.03). However, Trump supporters perceived discordant fake headlines as least accurate in the reason condition (*M* = 2.37) and as more accurate in the control (*M* = 2.44) and emotion (*M* = 2.54) conditions. Although these differences between conditions within partisan groups were not significant themselves, they suggest a potential interplay between thinking mode, partisanship, and political concordance. Notably, no evidence exists of either Clinton or Trump supporters perceiving concordant fake headlines as more accurate in the reason condition than in the emotion condition, which is unexpected under the motivated reasoning account.

#### Some evidence of interaction between condition, type of news, and study

To account for variation between experiments in our analyses, we fit a linear mixed model with condition, type of news, and study as fixed effects, allowing for all interactions. Experiment 2 served as our reference level for study. We included random intercepts by item and by participant nested by study as random effects. We were unable to include random slopes, as no random slopes model was able to converge. We found a joint significant interaction between condition, type of news, and study, *F*(4, 37,541.93) = 3.00, *p* = 0.017. This joint significant interaction appeared to be driven by the interaction between the reason condition, type of news, and experiment 4 (*p* = 0.001). Since experiment 4 utilized a different online platform (Lucid) than the other three experiments (MTurk), we fit a model replacing study with platform as a fixed effect. MTurk was the reference level platform. In this model, we were able to include random slopes by item for the interaction between condition and platform, as well as random slopes for type of news for participants nested by studies. With random slopes, we did not find a significant joint interaction between platform, condition, and type of news, *F*(2, 35.65) = 2.32, *p* = 0.113. The interaction between the reason condition, type of news, and platform was only marginally significant (*p* = 0.050). Taken together, these analyses suggest some evidence of a three-way interaction among study, type of news, and condition. As a result, we performed two separate versions of our main linear mixed-effects analysis looking at the relationship between accuracy, condition, and type of news: one with only our data from experiments 1 through 3 (MTurk) and one with the data from experiment 4 (Lucid). We found that the MTurk-specific results are similar to the results from our aggregated analyses, except the effects are even stronger: a significant effect of condition on fake news, *F*(2, 88.12) = 5.62, *p* = 0.005, and a significant interaction between condition and type of news, *F*(2, 66.37) = 4.83, *p* = 0.011, were observed. Conversely, our results from only the Lucid experiment were essentially null, with no condition effects. The results of these analyses are presented in the Additional file 1. Our Additional file 1 also include analyses assessing differences in adherence to our causal manipulations across experiments, in which we find adherence to be significantly lower in experiment 4 (Lucid) than in experiments 2 and 3 (MTurk). These results provide tentative evidence that lower adherence to our manipulations on Lucid may explain our null effects on Lucid in experiment 4.

## General discussion

Our results suggest several conclusions about the roles of emotion and reason in fake news perception. First, our findings from Study 1 indicate that momentary emotion, regardless of the specific type or valence of emotion, is predictive of increased belief in fake news and decreased discernment between real and fake news. Our results also suggest that emotion is specifically associated with belief in fake news. Therefore, rather than assessing how specific emotions impact perceptions of fake news, perhaps first assessing how emotion, in general, impacts belief in misinformation is best.

Second, our results from Study 2 further suggest clear correlational and experimental evidence that reliance on emotion increases belief in fake news. We found a positive association between self-reported use of emotion and belief in fake news, and that the more participants relied on emotion over reason, the more they perceived fake stories as accurate. Our manipulation also revealed causal evidence showing that inducing reliance on emotion results in greater belief in fake news compared to both a control and a condition where we induced analytic, logical thinking. Indeed, perhaps this study’s most notable finding is that reliance on emotion increases accuracy ratings of fake news relative to reliance on reason and relative to a control.

Our findings also provide some tentative evidence that the effect of emotion on perceptions of accuracy is specific to fake news. We found a significant correlational interaction between self-reported use of emotion and type of news headline (fake, real), suggesting that heightened reliance on emotion decreases people’s ability to discern between real and fake news. Our correlational analyses also showed that use of emotion was unrelated to real news accuracy perceptions. Additionally, we found no experimental effect of thinking mode on real news accuracy ratings. Although we only found a marginal overall interaction between condition and type of news headline, the interactions with type of news were significant when comparing emotion vs. control and emotion vs. reason; and the overall interaction was significant when consider the MTurk experiments (no manipulation effects at all were observed on Lucid). This tentatively suggests that inducing emotional thinking using a simple induction manipulation may impair the ability distinguish fake news from real, although further work is required.

Furthermore, the current studies suggest that belief in fake news is driven notably by over-reliance on emotion, relative to a simple lack of analytic reasoning. Use of reason was unrelated to fake news accuracy perceptions, and no difference was observed in accuracy perception between our experimental reason condition and the control condition. Therefore, emotion may be actively and uniquely promoting heightened belief in fake news relative to a baseline condition, and heightened reliance on emotion appears to be underlying susceptibility to fake news above and beyond a simple lack of reasoning.

Our evidence builds on prior work using the Cognitive Reflection Test (i.e., a measure assessing the propensity to engage in analytic, deliberative thinking; CRT; Frederick 2005), demonstrating a negative correlational relationship between CRT performance and perceived accuracy of fake news and a positive correlational relationship between CRT performance and the ability to discern fake news from real news (Pennycook and Rand 2019a). Beyond these correlational results, the current studies provide causal evidence that inducing heightened reliance on emotion increases susceptibility to believing fake news and tentatively suggest that increasing emotional thinking hinders media truth discernment.

Furthermore, our findings provide further evidence against the motivated account of fake news perception. Whereas the motivated account would predict analytic reasoning to increase ideologically motivated belief of politically concordant fake news (see Kahan 2017), our results show no interaction between condition and concordance. We find no evidence suggesting that people utilize ideologically motivated reasoning to justify believing in fake news; rather, people appear to believe fake news if they rely too heavily on intuitive, emotional thinking. The motivated account would also predict analytic thinking to justify greater belief in concordant real news. However, we do not find a statistically significant association between relative use of reason and perceived accuracy of concordant real news. Our findings support the classical account of fake news perception, which posits that a failure to identify fake news stems from some combination of a lack of analytic, deliberative thinking and heightened reliance on emotion. Therefore, the mechanism by which individuals fall prey to fake news stories closely resembles how people make mistakes on questions such as the bat-and-ball problem from the CRT; that is, people mistakenly “go with their gut” when it would be prudent to stop and think more reflectively. Just as the bat-and-ball problem has an intuitive, albeit wrong, answer, evidence suggests that people have an intuitive truth bias (see Bond and DePaulo 2006), and thus, analytic reasoning aids in overcoming such intuitions in some contexts. Indeed, an abundance of evidence suggests that individuals assume they are being informed of the truth and are bad at identifying lies and misinformation (e.g., Bond and DePaulo 2006; Levine et al. 1999). This suggests that an over-reliance on intuition—and, specifically, having a reflexively open-minded thinking style (Pennycook and Rand 2019c)—is likely to result in people being more susceptible to believing fake news. As we find, inducing emotional, intuitive reasoning does in fact increase the propensity to believe fake news stories.

Our findings have important practical implications. If emotional, nondeliberative thinking results in heightened belief of fake news, then the extent to which social media platforms bias people to think with emotion over reason may contribute to the viral success of fake news. Indeed, sentiment analysis of fake news articles reveal that fake news tends to contain increased negative emotional language (Zollo et al. 2015; Horne and Adali 2017). Even true yet emotionally stimulating content may result in people being biased to think with emotion instead of reason. Further applied research into how social media platforms may separately display non-news related, yet emotionally provocative, content and news articles may provide insight into how to prevent inducing emotional thinking in individuals online, thereby potentially decreasing general susceptibility to fake news.

### Limitations

Several potential limitations have been identified in the current research. First, the induction manipulation used across all four experiments was somewhat heavy-handed, and therefore, experimenter demand effects may be present. Future work should investigate whether similar patterns hold with alternative manipulations.

Second, although we find that reliance on emotion increases overall accuracy ratings of fake news, most individuals still consider fake news stories overall as more likely to be false than true. Thus, although reliance on emotion promotes belief in fake news overall, for a large proportion of participants, such reliance did not promote belief to the extent that participants found fake news stories to be more likely true than false. However, even incremental increases in belief (or reductions in disbelief) may contribute to greater long term belief (e.g., through repeated exposure; Pennycook et al. 2018).

Third, the classical account purports that analytic reasoning aids in overcoming intuitions such as automatic belief in false headlines. However, in the current research, we did not find evidence that inducing reason improves perceived accuracy of fake news or discernment between real and fake news relative to the control. Rather, we found that inducing intuitive, emotional thinking increased perceived accuracy of fake news. Therefore, susceptibility to fake news appears to be more about increased reliance on emotion rather than decreased analytic thinking. One potential explanation for why our induction of analytic thinking did not improve perceptions of fake news or discernment between real and fake news relative to the control is that participants in the control condition already may have been relying generally more on reason than emotion. This is supported by our manipulation check data, which suggests that people in the emotion condition used emotion relatively more than reason, whereas people in the control and reason conditions used reason relatively more than emotion. Such findings are also consistent with literature suggesting that, on average, fake news does not make up a large proportion of people’s media diets but rather is particularly consumed and shared by specific political and demographic groups (Guess et al. 2019, 2020). Our results are largely consistent with the general idea that fake news belief and consumption may be driven by a small share of individuals sharing specific traits—one of which may be extremely heightened reliance on emotion. Therefore, one potential avenue for future research may be investigating manipulations aimed at reducing reliance on emotion while consuming news specifically for individuals with heightened susceptibility to fake news.

Fourth, fake news is often aimed at eliciting high emotionality (Bakir and McStay 2018; Horne and Adali 2017) and specific emotions such as moral outrage (e.g., Crockett 2017). However, our current work does not specifically assess the relative emotionality of fake news and real news in the context of accuracy assessments. Indeed, a key feature of fake news may be that it is more emotionally provocative than real news. Therefore, our current research does not control for the arousal or valence of headlines across real and fake stimuli. Instead, the current studies focus on the individual’s experience of and reliance on emotion while making media accuracy judgments. An examination of whether heightened reliance on emotion promotes increased belief in fake news because of the increased emotionality of fake news headlines themselves or whether an increased reliance on emotion promotes belief in fake news due to increased gullibility or susceptibility to inaccurate information regardless of the intrinsic emotional arousal or valence of such content is beyond the scope of this study. To reiterate, whether similar results would be found if fake news stimuli were adjusted to have the same emotional content as our real news stimuli remains unclear. An interesting and important future research direction would be to assess the interaction between emotional processing and the emotional content of fake and real news. Nonetheless, our results from Study 2 still suggest that increased reliance on emotion in particular increase belief in fake news headlines as they would appear in a real world setting, such as on social media.

Fifth, our assessment of the relationship between emotion and news accuracy judgments does not consider the precise mechanisms by which specific emotions may influence ratings of news accuracy. Although we find in Study 1 that most emotions measured by the PANAS are associated with increased belief in fake news and decreased ability to discern between real and fake news, we cannot speak to whether the mechanisms behind these relationships are uniform or vary between emotions. A number of studies detail how different emotions are associated with different processing patterns; for instance, positive emotions may facilitate assimilative processing (i.e., changing external information to fit internal representations), whereas negative emotions may be associated with accommodative processing (i.e., changing internal representations to fit external information; see Fiedler and Beier 2014; Bohn-Gettler 2019). However, other models of emotional processing posit that both positive and negative emotions may place limitations on cognitive resources if experiencing such emotions is part of a semantic network (Meinhardt and Pekrun 2003). Furthermore, even more complex relationships between emotion and cognition may exist and explain our results; for instance, the same emotion may promote different judgments depending on the appraisal of that emotion (e.g., pleasantness/unpleasantness of confidence/doubt appraisal; see Briñol et al. 2018). Although we find that both positive and negative emotions are associated with greater belief in fake news, whether uniform or distinct emotional information processes and appraisals drive these results is unclear.

Sixth, our analyses do not examine the role of trait-based emotion in news accuracy judgments and belief in fake news. Emotions and affective responses have been found to be relatively stable over time (Diener and Larsen 1984), and these stable emotional states thus may reflect general affective personality traits. In our current work, we assess the role of momentary mood states (Study 1) and emotional processing (Study 2) on belief in fake news. However, we do not measure or manipulate trait-based emotions. Future research may examine how trait-based emotions may impact who falls for fake news.

Seventh, our analyses rely primarily on a convenience sample of online Mechanical Turk workers (experiments 1–3). Although previous work has shown that Amazon Mechanical Turk is a reasonably reliable resource for research on political ideology (Coppock 2019; Krupnikov and Levine 2014; Mullinix et al. 2015), our samples were not nationally representative and our political ideology comparisons should be interpreted with this in mind. However, when assessing the causal role of reason and emotion in perceiving fake news accuracy, obtaining a nationally representative population may not be as important as sampling from groups of people who are frequent internet and social media users and therefore likely encounter fake news stories more regularly. Thus, Mechanical Turk may be an even more appropriate resource than a nationally representative sample. Nevertheless, how our findings may generalize to different populations is unclear. In experiment 4, which utilized a more nationally representative sample via Lucid, we found no effect of condition on fake news perception or on media truth discernment. However, this was not a precisely estimated null, as it was also not significantly different from the overall estimate. Additionally, the null effect may have been caused by Lucid participants being less attentive than MTurkers, rather than due to their differential demographic characteristics, as Lucid participants are perhaps less professionalized than the MTurk population (Coppock and McClellan 2019). Indeed, we find that adherence to our emotion and reason manipulations is significantly lower in study 4 (Lucid) than in studies 2 or 3 (MTurk). However, whether the manipulation used in our study is effective across samples from different online recruitment platforms remains unclear. Future work should identify whether the effects we found in our MTurk data generalize to other platforms.

Finally, our experiments used only a small subset of all contemporary fake and real news headlines. Although these headlines were selected to be representative of fake and real news headlines in general, further research is required to ascertain how our findings would generalize to different headlines or to different displays of headlines other than the Facebook news article format.

## Conclusion

Dictionary.com recently named “misinformation” its 2018 word of the year and defined it as “false information that is spread, regardless of whether there is intent to mislead.” The online dissemination of misinformation and fake news is a troubling consequence of our digital age, and the need for psychologists to develop an understanding of the cognitive mechanisms behind why people fall for misinformation and fake stories so commonly viewed online is critical. The current results show that emotion plays a causal role in people’s susceptibility to incorrectly perceiving fake news as accurate. Contrary to the popular motivated cognition account, our findings indicate that people fall for fake news, in part, because they rely too heavily on emotion, not because they think in a motivated or identity-protective way. This suggests that interventions that are directed at making the public less emotional consumers of news media may have promise in reducing belief in fake news.

## Supplementary information

**Additional file 1.** Additional file contains descriptive statistics and additional analyses

## Data Availability

All data and materials are available online at https://osf.io/gm4dp/?view_only=3b3754d7086d469cb421beb4c6659556.

## Footnotes

1 Here we conduct an exploratory analysis of data from a study originally designed to investigate the effects of political echo chambers on belief in fake news. For simplicity, we focus on the results of participants who were randomly assigned to the control condition of this study in which participants saw a politically balanced set of headlines (although the results are virtually identical when including subjects from the other conditions, in which most headlines were either favorable to the Democrats or the Republicans).

2 See Additional file 1: Table S1 for relevant descriptive statistics.

3 Our PANAS scale internal reliabilities for positive and negative emotion were both acceptably high and in line with prior findings (e.g., Watson et al. 1988); Cronbach’s *α*_positive_ = 0.916 and Cronbach’s *α*_negative_ = 0.906.

4 This model may also be compatible with the circumplex model of affect, which posits that all affective states arise from common neurophysiological systems (Posner et al. 2005). In particular, while different affective processes and emotions may vary by valence and arousal, a common cognitive system underlying all emotional states may yet uniformly impact emotional information processing relevant to forming accuracy judgments of fake news.

5 We used Clinton versus Trump because the first experiment was completed in April, 2017—which was shortly after the inauguration. This question was then used in all subsequent experiments to retain consistency.

6 See Additional file 1: Table S2 for descriptive statistics of relevant measures and variables.

7 Degrees of freedom calculated via joint significant tests within the lmer R package are computed using the Kenward–Roger degrees of freedom approximation; hence, the denominator degrees of freedom in our joint significance tests tend not to be integers.
