# Supplementary material for: Reliance on emotion promotes belief in fake news
Source: Cogn Res Princ Implic. 2020 Oct 7;5:47. doi: 10.1186/s41235-020-00252-3 (PMC7539247; doi:10.1186/s41235-020-00252-3)
Supplement: Supplementary file 1 — Additional file 1. Additional file contains descriptive statistics and additional analyses [file 41235_2020_252_MOESM1_ESM.docx]

**Supplemental Materials**

**Descriptive statistics of key variables and measures.**

Table S1. *Descriptive statistics for Study 1, including means and standard deviations for fake news accuracy ratings, real news accuracy ratings, partisanship, and all 20 PANAS items.*

| Variable | *Mean* | *SD* |
| --- | --- | --- |
| Fake News Accuracy Ratings | 1.62 | 0.47 |
| Real News Accuracy Ratings | 2.84 | 0.44 |
|  |  |  |
| Partisanship (Democrat) | 0.64 | 0.48 |
|  |  |  |
| Enthusiastic | 2.71 | 1.21 |
| Interested | 3.38 | 1.02 |
| Determined | 3.26 | 1.14 |
| Excited | 2.33 | 1.20 |
| Inspired | 2.51 | 1.20 |
| Alert | 3.54 | 1.03 |
| Active | 2.64 | 1.17 |
| Strong | 2.89 | 1.15 |
| Proud | 2.50 | 1.20 |
| Attentive | 3.77 | 0.92 |
| Scared | 1.32 | 0.73 |
| Afraid | 1.33 | 0.72 |
| Upset | 1.38 | 0.79 |
| Distressed | 1.52 | 0.86 |
| Jittery | 1.59 | 0.93 |
| Nervous | 1.57 | 0.93 |
| Ashamed | 1.31 | 0.74 |
| Guilty | 1.31 | 0.69 |
| Irritable | 1.58 | 0.87 |
| Hostile | 1.26 | 0.71 |

Table S2. *Descriptive statistics for Study 2, including means and standard deviations for fake and real news accuracy ratings (overall and by condition), as well as for partisanship.*

| Variable | *Mean* | *SD* |
| --- | --- | --- |
| Fake News Accuracy Ratings | 2.69 | 0.91 |
| Fake News Accuracy Ratings - Emotion | 2.75 | 0.92 |
| Fake News Accuracy Ratings - Control | 2.66 | 0.90 |
| Fake News Accuracy Ratings - Reason | 2.66 | 0.92 |
|  |  |  |
| Real News Accuracy Ratings | 4.22 | 0.73 |
| Real News Accuracy Ratings - Emotion | 4.22 | 0.74 |
| Real News Accuracy Ratings - Control | 4.20 | 0.73 |
| Real News Accuracy Ratings - Reason | 4.24 | 0.73 |
|  |  |  |
| Partisanship (Democrat) | 0.60 | 0.49 |

**Controlling for familiarity in correlational analyses.**

***Study 1.***

We performed several additional analyses as part of Study 1 in order to control for the possible effects of headline familiarity on accuracy judgments. We modified our main mixed effects models predicting accuracy from overall positive emotion and overall negative emotion. These mixed effects models predicted accuracy as our dependent variable, and included overall emotion (positive or negative), type of news headline, the interaction between emotion and type of news headline, headline familiarity (scaled; as assessed via pre-testing of headlines; see Pennycook & Rand, 2019a), and the interaction between headline familiarity and type of news headline. As random effects, we included random intercepts for item and participant, and random slopes for emotion and random slopes for the interaction between type of news headline and familiarity.

We found that even when controlling for headline familiarity, our results largely remained the same. Experiencing greater positive emotion was associated with greater belief in fake headlines, *b* = 0.14, *SE* = 0.02, *t*(87.25) = 6.54, *p* < .001. Furthermore, we found an interaction between experiencing positive emotion and type of news headline, such that discernment was worse for those experiencing more positive emotion, *b* = -0.12, *SE* = 0.02, *t*(31.13) = -5.58, *p* < .001. Furthermore, a joint significance test did not find an effect of experiencing positive emotion on belief in real news headlines (*p* = .578).

Our results were similar when looking at experience of negative emotions. Experiencing greater negative emotion was associated with greater belief in fake headlines, *b* = 0.18, *SE* = 0.02, *t*(89.29) = 7.59, *p* < .001. Additionally, we found an interaction between experiencing negative emotion and type of news headline, such that discernment was worse for those experiencing more negative emotion, *b* = -0.19, *SE* = 0.03, *t*(39.36) = -7.21, *p* < .001. A joint significance test did not find an effect of experiencing negative emotion on belief in real news headlines (*p* = .534).

Overall, these analyses suggest that even when controlling for news headline familiarity, we find that greater self-reported experience of momentary emotion, for both positive and negative emotion, is associated with greater belief in fake news and decreased discernment between fake and real news.

***Study 2.***

We also performed follow-up analyses for our correlational analyses from Study 2, controlling for headline familiarity. We first performed a mixed effects model predicting accuracy, with relative use of reason, type of news headline, the interaction between relative use of reason and type of headline, familiarity, and the interaction between type of news headline and familiarity as fixed effects. As random effects, we included random intercepts for items and participants nested by study, as well as random slopes for relative use of reason and the interaction between type of news headline and familiarity. Our results when controlling for familiarity remained largely consistent with our main findings. We again found that greater relative use of reason was associated with decreased belief in fake news headlines, *b* = -0.17, *SE* = 0.02, *t*(56.18) = -7.41, *p* < .001, and that there was a significant interaction between relative use of reason and type of news headline, such that greater relative use of reason was associated with increased discernment between real and fake news, *b* = 0.20, *SE* = 0.03, *t*(38.82) = 6.81, *p* < .001. A joint significance test found no effect of relative use of reason on accuracy ratings for real news stories, *p* = .251.

We again found different relationships with use of reason versus use of emotion. Performing similar mixed effects models as described above (except substituting relative use of reason for use of reason and use of emotion, respectively), we did not find that use of reason was associated with belief in fake headlines. Rather, we found that use of reason was associated with greater belief in real headlines (*p* < .001) and that use of reason interacted with type of news headline, such that those who used greater reason were better able to discern between real and fake news (*p* < .001). In contrast, we found that use of emotion was associated with greater belief in fake headlines (*p* < .001), and that there was a significant interaction between use of emotion and type of news headline, such that greater use of emotion decreased the ability to discern between real and fake news (*p* < .001). We did not find a relationship between use of emotion and belief in real headlines (*p* = .148).

Again, these results are all consistent with our main correlational findings from Study 2, suggesting that even when controlling for headline familiarity, we find that relative use of reason is associated with belief in fake headlines and decreased discernment between real and fake headlines. We also find that use of emotion is associated with belief in fake headlines, whereas use of reason is associated with belief in real headlines, even when controlling for headline familiarity.

**The role of adherence to manipulation on news accuracy perceptions.**

In order to investigate whether there were difference between participants in terms of how well they adhered to our manipulations, we investigated how the extent to which participants adhered to our manipulations impacted their headline accuracy ratings. We analyzed data from experiments 2, 3, and 4, which each contained ratings of self-reported use of reason and use of emotion, and we again combined these measures to establish a relative use of reason score (i.e., self-reported use of reason minus self-reported use of emotion). Next, we filtered for only participants who were in either the emotion or reason condition, omitting the control group for which adherence was not measured. Then, we defined ‘adherence’ as the extent to which self-reported relative use of reason aligned with the participant’s assigned condition – in particular, for participants in the reason condition, their adherence score was simply their relative use of reason, whereas adherence for participants in the emotion condition was the negation of their relative use of reason (i.e., relative use of emotion).

We then performed a linear mixed effects analysis predicting perceived news accuracy, with adherence, condition (emotion, reason), type of news headline, and all interactions as fixed effects (as well as a study dummy variable). As random effects, we included intercepts for headline items and participants nested by study, and we also included by-item random slopes for the interaction between adherence and condition, as well as by-nested participant random slopes for type of news headline.

This analysis yielded predictable results. We found that for participants in the emotion condition, greater adherence to our manipulation was associated with increased belief in fake news headlines, *b* = 0.05, *SE* = 0.02, *t*(90.02) = 2.87, *p* = .005. We also found a negative interaction term between adherence and the reason condition, such that greater adherence in the reason was associated with lower accuracy ratings for fake news headlines, *b* = -0.23, *SE* = 0.03, *t*(61.43) = -7.27, *p* < .001. These results suggest that adherence to our manipulations had predictable results on perceptions of fake news, such that greater adherence in the emotion condition was associated with increased belief in fake news, while greater adherence in the reason condition was associated with decreased belief in fake news. These results also suggest that accuracy ratings by individuals more attentive to our causal manipulations were perhaps more influenced by our emotion and reason conditions, supporting the causal validity of our experimental paradigm.

We also examined whether some participants adhered to our manipulations better than others. We found that adherence was slightly correlated with partisanship, such that Trump (rather than Clinton) supporters adhered slightly less to our manipulation – however, while this relationship was significant, it was also very small and unlikely to be practically significant, *r* = -0.01, *t*(45958) = -2.08, *p* = .038.

We also performed an ANOVA to assess whether adherence differed between experiments 2, 3, and 4. Here, we found that adherence significantly differed between experiments, *F*(2, 46037) = 2312, *p* < .001. In particular, as evident by follow-up Tukey tests, this was driven by adherence being significantly lower in experiment 4 (*M* = 0.49) than in experiment 2 (*M* = 1.77) or experiment 3 (*M* = 1.731.73), *p*s < .001. There was no difference in adherence between experiments 2 and 3, *p* = .180. This suggests that participants in Study 4 adhered less to our manipulation and were perhaps less attentive to our manipulation.

**Evidence of causal manipulation effect on news accuracy perception in MTurk experiments.**

We examined whether there was a condition effect on the perceived accuracy of fake and real news across experiments 1 through 3, which were all performed on Amazon Mechanical Turk (MTurk). We performed a linear mixed effects analysis of the relationship between perceived news accuracy, experimental condition (emotion, control, reason), and type of news headline. As fixed effects, we entered condition and type of news headline, with an interaction term. As random effects, we included intercepts for headline items and participants nested by study, and we also included by-item random slopes for condition and by-nested participant random slopes for type of news headline. The reference level for condition was ‘emotion’ and the reference level for type of news headline was ‘fake’. The results of this analysis are shown in Table S3.

Table S3. *Results of linear mixed effects analysis of accuracy by condition and type of news article, for experiments 1-3 (MTurk).*

|  | *beta* | *SE* | *df* | *\|t\|* | *p* |
| --- | --- | --- | --- | --- | --- |
| Intercept | 2.79 | .23 | 3.52 | 11.96 | .001 |
| Control (condition) | -0.14 | 0.05 | 72.27 | -3.20 | .002 |
| Reason (condition) | -0.12 | 0.04 | 49.11 | -2.70 | .009 |
| Real (headline truth) | 1.13 | 0.18 | 26.93 | 6.44 | < .001 |
| Control : Real | 0.14 | 0.06 | 46.87 | 2.47 | .017 |

**Emotion condition heightens belief in fake news in MTurk experiments.**

A joint significance test revealed a significant effect of condition on fake news accuracy perception, *F*(2, 88.12) = 5.62, *p* = .005. Fake news headlines were reported as significantly more accurate in the emotion condition as compared to the control condition (*p* = .002) and the reason condition (*p* = .009). A joint significance test of condition on real news accuracy perception did not show a significant effect, *F*(2, 66.42) = 1.89, *p* = .159.

**Significant effect of condition on discerning fake from real news in MTurk experiments.**

We next performed a joint significance test of the interaction between condition and news type. This revealed a significant interaction, *F*(2, 66.37) = 4.83, *p* = .011. The coefficients of our model show that media truth discernment, as indicated by the interaction between condition and news type, is greater in the control condition than in the emotion condition (*p* = .017), and also greater in the reason condition than in the emotion condition (*p* = .004). Therefore, it appears that there is a strong effect of condition on media truth discernment when looking only at the experiments performed on MTurk. However, the results of our aggregated data analysis, which includes experiment 4 (Lucid), suggest only a marginal effect on discernment.

**No condition effect on fake news or discernment in Lucid experiment.**

We next performed a nearly identical linear mixed effects analysis except selecting for data from experiment 4, which was performed on the platform Lucid. The only difference in the model itself was that random effects were accounted for by participant rather than participant nested by experiment, since this model only utilizes data from a single study. Unlike our results from collapsing across all four experiments and our results looking only at the MTurk experiments, this model revealed no condition effect on accuracy judgements of fake news, *F*(2, 166.12) = 0.65, *p* = .524. We again found no condition effect for real headlines, *F*(2, 55.07) = 0.56, *p* = .576. Finally, there was no condition effect on discernment, *F*(2, 61.22) = 0.30, *p* = .739. Taken together, these results suggest that the induction manipulation was ineffective on Lucid, and that there appears to be a notable difference between our results from MTurk and our results from Lucid, despite there only being a marginally significant interaction effect of platform on discernment.
